# Supplementary material for: Mapping SBiP1 protein-protein interactions in Symbiodinium microadriaticum CassKB8 using the Yeast Two-Hybrid assay and structural prediction
Source: PLoS One. 2026 Feb 6;21(2):e0340367. doi: 10.1371/journal.pone.0340367 (PMC12880724; doi:10.1371/journal.pone.0340367)
Supplement: S1 Table — Candidate sequences identified by the yeast two-hybrid screen and analyzed using BLAST against the NCBI nucleotide or protein databases. (PDF) [file pone.0340367.s002.pdf]

Supplementary Table 1. Top BLAST hits for each SBiP1-interacting candidate. Each candidate sequence identified by the yeast two-hybrid screen was analyzed using BLAST against the NCBI nucleotide or protein databases. The top-scoring hit per candidate was selected based on the highest total alignment score. For each entry, the matched protein description, source organism, E-value, percentage identity, query coverage, and GenBank accession number are reported. These annotations support the initial functional classification of the interactors and help validate their potential biological roles.

	Top BLAST Hit Description	Organism	Query Coverage	E-value	% Identity	Accession
**C1**	Hsp70 [*Crypthecodinium cohnii*]	*Crypthecodinium cohnii*	69%	0	94.49	AAM02973.2
**C2**	Peroxisomal multifunctional enzyme type 2 [*Symbiodinium microadriaticum*]	*Symbiodinium microadriaticum*	67%	2.98e-49	77	OLP96676.1
**C3**	TSA: *Symbiodinium* sp. A4 strain CCMR0100 isolate 043D10 comp9992_c0_seq1, transcribed RNA sequence	*Symbiodinium* sp. A4	74%	0	91.43	GFPM01011551.1
**C4**	Putative methyltransferase TARBP1 [*Symbiodinium microadriaticum*]	*Symbiodinium microadriaticum*	81%	3e-132	85.08	OLQ14198.1
**C5**	hSD17B4 [*Symbiodinium* sp. KB8]	*Symbiodinium* sp. KB8	47%	1e-56	99.1	CAE7477333.1
**C6**	Unnamed protein product [Polarella glacialis]	*Polarella glacialis*	40%	6e-40	94.44	CAE8603653.1
**C7**	*Symbiodinium kawagutii* strain CCMP2468 clone SymkaSLLR3-204, partial sequence	*Symbiodinium kawagutii*	84%	5e-105	81.39	KC937275.1
**C8**	gTPBP1 [*Symbiodinium necroappetens*]	*Symbiodinium necroappetens*	53%	5E-43	100	CAE7191322.1
**C9**	*Symbiodinium microadriaticum* subsp. *microadriaticum* isolate CassKB8 translation elongation factor 1 alpha-like protein (EF-1alpha) mRNA, complete cds	*Symbiodinium microadriaticum subsp. microadriaticum*	99%	0	96.66	KT183028.1
**C10**	HSD17B4 [*Symbiodinium* sp. KB8]	*Symbiodinium* sp. KB8	66	4e-57	99.1	CAE7477333.1
**C11**	TSA: *Symbiodinium* sp. CCMP2430, TRINITY-DN46071-c0-g1-i1, transcribed RNA sequence	*Symbiodinium* sp. CCMP2430	99	0	88.22	HBTH01072961.1
