# Supplementary material for: Mapping SBiP1 protein-protein interactions in Symbiodinium microadriaticum CassKB8 using the Yeast Two-Hybrid assay and structural prediction
Source: PLoS One. 2026 Feb 6;21(2):e0340367. doi: 10.1371/journal.pone.0340367 (PMC12880724; doi:10.1371/journal.pone.0340367)
Supplement: S2 Table — The eleven SBiP1-interacting candidates predicted using AlphaFold2 are shown. (PDF) [file pone.0340367.s003.pdf]

Supplementary Table 2. Summary of structural quality metrics for SBiP1-interacting candidates. The eleven SBiP1-interacting candidates predicted using AlphaFold2 are shown. pLDDT (predicted Local Distance Difference Test) scores reflect model confidence at the residue level.

	ANNOTATION	SEQUENCE LENGTH (AA)	MEAN PLDDT	CONFIDENCE LEVEL	STRUCTURAL DOMAINS	PREDICTED INTERACTION SURFACE
**C1**	HSP70	648	99.78	Very high	Canonical ATPase and SBD domains	Hydrophobic cleft (substrate-binding domain)
**C2**	POX18	726	99.78	High	Multidomain: enoyl-CoA hydratase + HAD	Hydrophobic patches near HAD-like domain
**C3**	Unknown	189	38.18	Intermediate	Alpha-helical, globular	Basic and nonpolar residues exposed
**C4**	TARBP1	1052	53.47	High	SAM-binding methyltransferase fold	Flexible loops around SAM pocket
**C5**	POX18	726	50.37	High	Multidomain: enoyl-CoA hydratase + HAD	Hydrophobic patches near HAD-like domain
**C6**	MAP2B	380	89.72	High	Compact M24 peptidase-like	Shallow cavity in C-terminal domain
**C7**	GTPBP1	520	75.33	High	GTPase fold, alpha/beta	Polar loop adjacent to GTP pocket
**C8**	WRAP73	950	76.17	Intermediate	Beta-propeller WD40 repeats	Distributed surface, scaffold-like
**C9**	EFL	423	79.68	High	EF-Tu GTPase-like domain	Hydrophobic loop near GTP site
**C10**	POX18	726	96.22	High	Multidomain: enoyl-CoA hydratase + HAD	Hydrophobic patches near HAD-like domain
**C11**	TARBP1	1052	48.9	Intermediate	SAM-binding methyltransferase fold	Flexible loops around SAM pocket
