# Supplementary material for: Mapping SBiP1 protein-protein interactions in Symbiodinium microadriaticum CassKB8 using the Yeast Two-Hybrid assay and structural prediction
Source: PLoS One. 2026 Feb 6;21(2):e0340367. doi: 10.1371/journal.pone.0340367 (PMC12880724; doi:10.1371/journal.pone.0340367)
Supplement: Attachment — Submitted filename: Response to Reviewers.pdf [file pone.0340367.s005.pdf]

Response to Reviewers:

We wish to thank both reviewers for their keen observations which have significantly improved the manuscript.

Reviewer #1:

I recommend the paper for publication following the minor corrections detailed below.

Query: The Y2H assay is a high throughput and low cost way to investigate the interactome and a good method for organisms like Symbiodiniaceae that are notoriously hard to genetically alter. However, due to the high rate of false positives in the Y2H (up to 50%; Von Mering et al., 2002), the authors should make it clear to the reader that further work is needed to confirm the findings e.g., refer to the interacting proteins found in this study as candidates (which the authors have done in the introduction) and explicitly say that the study is an "initial screen" of the SBiP1 interactome. Furthermore, the authors could elaborate more on the weaknesses of the Y2H assay in the limitations section of the discussion. This could include the authors suggesting another method to corroborate the findings from the Y2H assay, with two/three methods investigating protein interactions increasing the accuracy of correctly identifying the interacting proteins (Von Mering et al., 2002).

Response: We thank the reviewer for this valuable suggestion. The manuscript was revised to explicitly describe the study as an initial yeast two-hybrid (Y2H) screen of the SBiP1 interactome. The limitations of the Y2H approach are now further considered in a new section added to the discussion (lines 526-544) "Limitations of the Y2H approach and qPCR observations", emphasizing the system's high false-positive rate and the need for complementary validation methods for this approach such as co-immunoprecipitation, affinity purification–mass spectrometry, and proximity labeling (lines 526-533) as well as for the qPCR (lines 534-544). Additionally, a sentence in the Abstract was placed (lines 19-20) to read "we performed an initial yeast two-hybrid screening…", and in the Introduction (lines 112-114) to read "We aimed to perform an initial Y2H screening to identify putative SBiP1 interactors…". Finally, we clarified in the results (lines 362-365) that, prior to conducting gene expression analyses, two representative candidates (HSP70 and POX18) were reconfirmed by additional yeast mating assays to further ensure the specificity of these interactions. A statement mentioning this limitation (lines 555-557) was also added in "Concluding Remarks".

Query: Line 478-485 (now 495-503)- Confused with this paragraph:
Figure 4 and 5 show transcripts do change in abundance over time with growth, and the results also say that both Pox18 and Hsp70 exhibit "significant temporal modulation".
Statement made on line 481 contrasts with sentence above - do you mean "regulated through post-translational mechanisms"?
Again, the sentence beginning on line 483 seems to contrast to the patterns seen in the figures.

Response: We thank the reviewer for this observation. The text discussing the expression dynamics of *HSP70* and *POX18* has been revised for clarity and consistency with the data. On lines 495-503, we now state that both genes exhibit moderate but significant temporal modulation in transcript abundance, mention their maximum levels of expression, and discuss that the magnitude of these changes suggests that regulation likely occurs primarily at the post-translational level.

Query: Line 486 (now 505)- From looking at the graph for CHX, although the transcript expression is only significantly different in Hsp70 control vs CHX treatment, the other transcripts show similar patterns (higher expression after CHX treatment vs control). I don't think from these findings you can conclude that Hsp70 is under feedback transcript stabilisation versus SBiP1 or POX18 are transcriptionally stable and not linked to active translation. Maybe if more replicates were included all the genes would show the same significance. Authors could reword to say "Hsp70 shows a weak significant increase" and mention that the other transcripts show exactly the same pattern, but it doesn't meet significance.

Response: We appreciate this constructive comment. The section has been modified on lines 505-507, to state that *HSP70* shows a weak but significant increase in transcript levels, while *SBiP1* and *POX18* follow the same upward trend without reaching statistical significance. The part of a "feedback stabilization" mechanism was removed, and the paragraph now indicates that translation inhibition may cause a general, modest accumulation of transcripts rather than a gene-specific regulatory effect.

Query: Line 503 (now 520)- not sure if the findings confirm that SBiP1 is a "central hub" due to Y2H assay limitations - reword as the authors need extra confirmation of protein-protein interactions before making this statement

Response: We have modified this statement accordingly and now only suggest an initial proposed role as regulatory hub for SBiP1 (lines 520-521), and on line 552-553, we now state that "our study suggests that SBiP1 may function as a potential regulatory hub in the ER homeostasis network" rather than as a confirmed central hub. We also acknowledge that additional experimental validation beyond Y2H is required to confirm these interactions and establish a SBiP1 integrative role.

Query: Typo in Figure 5 - should say "Hsp70" in graph title not "Hxp70".

Response: The typo in Figure 5 has been corrected ("Hsp70" instead of "Hxp70").

Query: Line 382 (now 391)- the text says that the expression of Hsp70 peaked at day 12 but figure 5 shows it peaking at day 6. The numbers in the text for change in expression on day 12 and 6 also don't match those on the figure - looks to be around 6.5-fold for day 6 and 6.25-fold for day 12.

Response: We appreciate the reviewer's observation. The text in the Results section has been revised (lines 391-393) to accurately reflect the data shown in Figure 5 (also same response to second query above), now indicating that *HSP70* expression increased sharply at day 6 and remained elevated at day 12 before decreasing toward baseline levels at later time points.

Reviewer #2: The manuscript is generally well written; however, there are several areas where improvements are needed.

Query: In the Abstract, acronyms should be defined before they are used. For example, ER appears without being defined anywhere in the manuscript.

Response: We thank the reviewer for noting this oversight. The acronym "ER" has now been defined as "endoplasmic reticulum (ER)" on line 67 in the Introduction section and checked for

consistency throughout the manuscript.

Query: Additionally, the figures could be improved by enhancing their resolution, increasing clarity of labels, and ensuring that all elements are consistently formatted and redundant info removed (e.g., parts in brackets for Figures 2-6).

Response: We appreciate the reviewer's feedback. All figures (1–6) were reformatted to improve resolution and label clarity, ensuring consistent font size, color palette, and axis style. Redundant elements in figure titles were removed for a cleaner and more uniform presentation.

Query: Can author add limitation regarding the way how gene expression was assessed as using only one reference genes may not be sufficient. Why was this specific reference gene SmicEF1-α used? Did you consider other genes? What was validation process here?

Response: We added in the text extensive background to support the use of *EF1-alpha* as the reference gene above any others due to its adequate performance in qPCR assays in numerous instances of species, developmental stages and culture conditions including microalgae and Symbiodiniaceae (references 35-42). The mentioned corresponding references have been added to the text in the Methods (lines 226-227), Results (line 323), and Discussion (536-537) sections. Furthermore, we have also provided additional support for the validity of the data on lines 490-492 and 540-544, by mentioning our previous results using western immunoblotting [reference 40], where we found constant SBiP1 protein expression under either dark or light conditions and which are fully coincident with part of the results from this study (Fig. 3).
