# Supplementary material for: Mapping SBiP1 protein-protein interactions in Symbiodinium microadriaticum CassKB8 using the Yeast Two-Hybrid assay and structural prediction
Source: PLoS One. 2026 Feb 6;21(2):e0340367. doi: 10.1371/journal.pone.0340367 (PMC12880724; doi:10.1371/journal.pone.0340367)
Supplement: Attachment — Submitted filename: Response_to_Reviewers_auresp_2.pdf [file pone.0340367.s006.pdf]

Response to Reviewer #1.

Reviewer #1: The authors have nicely addressed the points detailed in the previous review round. I have one more comment after re-reading the manuscript that would be nice for the authors to elaborate on and clarify their conclusions.

Paragraph starting line 389 and the sentences 498-503. The authors say that the "magnitude of the fluctuations" of the changes in gene expression in Hsp70 and Pox18 implies that they may be regulated at the post-translational level. I wasn't too sure whether the authors are implying the change in gene expression is low? However, after looking at the graphs, the change in expression seems quite high for Symbiodiniaceae (up to 4.21 fold for Pox18 and 6.32 fold for Hsp70) versus other papers that say most genes show minimal changes in expression (e.g., Gierz et al. 2017) and that's why other papers have hypothesised that most regulation may be done at the post-translational level. From the data presented here in this current study, it seems like the opposite, where Hsp70 and Pox18 may have high regulation at the transcript level. The authors do say this in lines 393-395 but the sentence following (line 395-398) contradicts this statement. Please could the authors clarify this.

Response:

Thank you for raising this observation. We agree that the observed fold-changes (up to ~6.3× for *HSP70* and ~4.2× for *POX18*) in expression are relatively high compared to most Symbiodiniaceae genes, which typically show minor variation in transcript levels.

To address this query, we have revised the text in both the Results (lines 396-401) and Discussion (lines 501-508) sections. We have added that previous reports document low-level transcriptional changes in Symbiodiniaceae as noted by the reviewer (Gierz et al. 2017; reference [3]) and that our observed data suggest a transcriptional component in the regulation of *HSP70* and *POX18*, while post-translational mechanisms likely act also in parallel to fine-tune functional regulation.
